# Thousands of trait-specific KASP markers designed for diverse breeding applications in rice (*Oryza sativa*)

**DOI:** 10.1093/g3journal/jkae251

**Published:** 2024-11-01

**Authors:** Katherine Steele, Mark Quinton-Tulloch, Darshna Vyas, John Witcombe

**Affiliations:** School of Environmental and Natural Science, Bangor University, Bangor, Gwynedd LL57 2UW, UK; School of Environmental and Natural Science, Bangor University, Bangor, Gwynedd LL57 2UW, UK; LGC BioSearch Technologies, Units 1 and 2, Trident Industrial Estate, Pindar Road, Hoddesdon, Herts EN11 0WZ, UK; School of Environmental and Natural Science, Bangor University, Bangor, Gwynedd LL57 2UW, UK

**Keywords:** genetic resources utilization, InDel, SNP, trait selection

## Abstract

This study aimed to broaden applicability of KASP for *Oryza sativa* across diverse genotypes through incorporation of ambiguous (degenerate) bases into their primer designs and to validate 4,000 of them for genotyping applications. A bioinformatics pipeline was used to compare 129 rice genomes from 89 countries with the *indica* reference genome R498 and generate ∼1.6 million KASP designs for the more common variants between R498 and the other genomes. Of the designs, 98,238 were for predicted functional markers. Up to 5 KASP each for 1,024 breeder-selected loci were assayed in a panel of 178 diverse rice varieties, generating 3,366 validated KASP. The 84% success rate was within the normal range for KASP demonstrating that the ambiguous bases do not compromise efficacy. The 3,366-trait-specific marker panel was applied for population structure analysis in the diversity panel and resolved them into 4 expected groups. Target variations in 13 genomes used for designs were compared with the corresponding KASP genotypes in different accessions of the same 13 varieties in the diversity panel. There was agreement for 79% or more markers in 12 varieties; 10 having agreement >88%. One variety, a selection from a landrace, had only 46.5% marker agreement. Breeders can search for the validated KASP and more than a million so-far untested designs in three reference genomes (including Niponbare MSU7) with a search tool, that includes designs in proximity to previously published microsatellite markers, and retrieve target variations for 129 rice genomes plus their genomic locations with ±25 bp flanking sequences.

## Introduction

Public and private sector rice breeders require efficient markers for selective breeding to enable global rice production to sustainably increase. In many rice-dependent regions, the benefits of genomic markers for rice improvement are still to be fully explored (Chakraborti *et al*. 2021); yet, it has been demonstrated that genomics-derived molecular markers can be effectively integrated into traditional rice breeding programs (Cobb *et al*. 2019).

Rice genome resequencing and bioinformatics have previously been used to identify large numbers of useful genomic DNA variants—single nucleotide polymorphism (SNP) and insertion/deletion (InDel)—that can be of use to breeders (Pariasca-Tanaka *et al*. 2015; Cheon *et al*. 2018; Sandhu *et al*. 2022). The bioinformatics skills necessary to identify suitable assays represent a high technology barrier for some breeders. Searchable databases for genomic variants exist for all major cereals (Thudi *et al*. 2021). Many rice researchers and breeders use IRRI's Rice SNP-Seek Database (snp-seek.irri.org; Mansueto, *et al*. 2017) and the Chinese Rice VarMap (ricevarmap.ncpgr.cn; Zhao *et al*. 2015). Despite this wide availability of variants associated with genes and quantitative trait loci (QTLs), there has been limited uptake of genomic breeding tools by public sector and small-scale rice breeders. Many types of marker technologies have been developed for SNP genotyping, but not all can be adopted readily in existing laboratories. Some marker technologies are less transferable to marker-assisted selection (MAS) applications than others. Some SNP panels are population specific (Heslot *et al*. 2013) and markers targeting suitable variants might not be readily identifiable for selection of traits in specific crosses (Makhoul *et al*. 2020). Such issues hinder adoption of new marker technologies, hence, microsatellite (SSR) markers developed in the 1990s remain popular among rice breeders, largely due to the readily searchable information in the Gramene markers database (archive.gramene.org/markers/; Liang *et al*. 2008).

KASP is PCR-based genotyping technology ideally suited for small- or large-scale genotyping applications. However, it is not always easy for breeders to locate useful information about suitable KASP markers for their uses. This is partly because KASP is a patented technology of LGC BioSearch Technologies (LGC) and primer sequences constitute intellectual property (IP). This study resolves this limitation by using the KASP genomic locations and ±25 bp flanking sequences so that breeders have sufficient information to either order them from LGC or use the location and sequence information to design their own primers.

There are considerable benefits to be gained in moving a marker-assisted breeding program from SSRs to a KASP-based approach (Steele *et al*. 2018; Kim *et al*. 2021). High-throughput KASP offer greater cost-effectiveness than SSRs but have similar levels of flexibility and can be used for population studies (e.g. Shikari *et al*. 2021), linkage mapping (e.g. Qureshi *et al*. 2018), and MAS (Kim *et al*. 2021).

There are 2,055 KASP in LGC's original Rice assay search tool, developed by Generation Challenge Program (Pariasca-Tanaka *et al.* 2015). Separately, Lee *et al*. (2022) developed 2,565 KASP from the C7AIR SNP array. KASP are increasing in popularity for QTL analysis and MAS in a range of agriculturally important species (Cheon *et al*. 2018; Kante *et al*. 2018; Devran and Kahveci 2019; Paudel *et al.* 2019; Van Inghelandt *et al*. 2019; Kaur *et al.* 2020; Zhang *et al*. 2020; Zhao *et al*. 2021). A valid concern for QTL mapping with KASP assays is that they may miss many rare variations or common alleles absent from the samples used to develop the assays (Scott *et al*. 2020). This can be overcome by sequencing the parents used by breeders for their crosses under study for de-novo marker development. However, this step is not practical for many rice breeders, so the goal of this study was to develop off-the-shelf SNP and InDel markers that should be representative across *Oryza sativa*.

KASP technology was selected for this study, following a successful feasibly study with breeders from Nepal who incorporated KASP-derived genotyping data into their breeding programs. The feasibility study only sampled 9 genomes to identify variants and design KASP primers (Steele *et al*. 2018). This study used a comparison of 129 genomes to identify suitable target variants (SNP or InDel) and also identified SNP variation occurring in the flanking regions of each target variant so that this information could inform the incorporation of degenerate bases in primers. The addition of degenerate bases was predicted to extend the efficacy of the resultant KASP assays and thereby broaden their applicability in different varieties. In this study, we selected 4,000 KASP assays of potential value for precision trait selection and applied them in genotyping an independently obtained diverse rice population. The study aimed to (i) determine if the number or location of ambiguous bases differed between successful and failing designs, (ii) demonstrate the utility of a ∼4 K KASP panel for resolving population structure, and (iii) provide a database of information for all the new KASP designs including their proximity to existing SSRs and C6IAR SNPs that can help breeders select them for different applications.

## Materials and methods

### Rice genome data

**Fig. 1.**
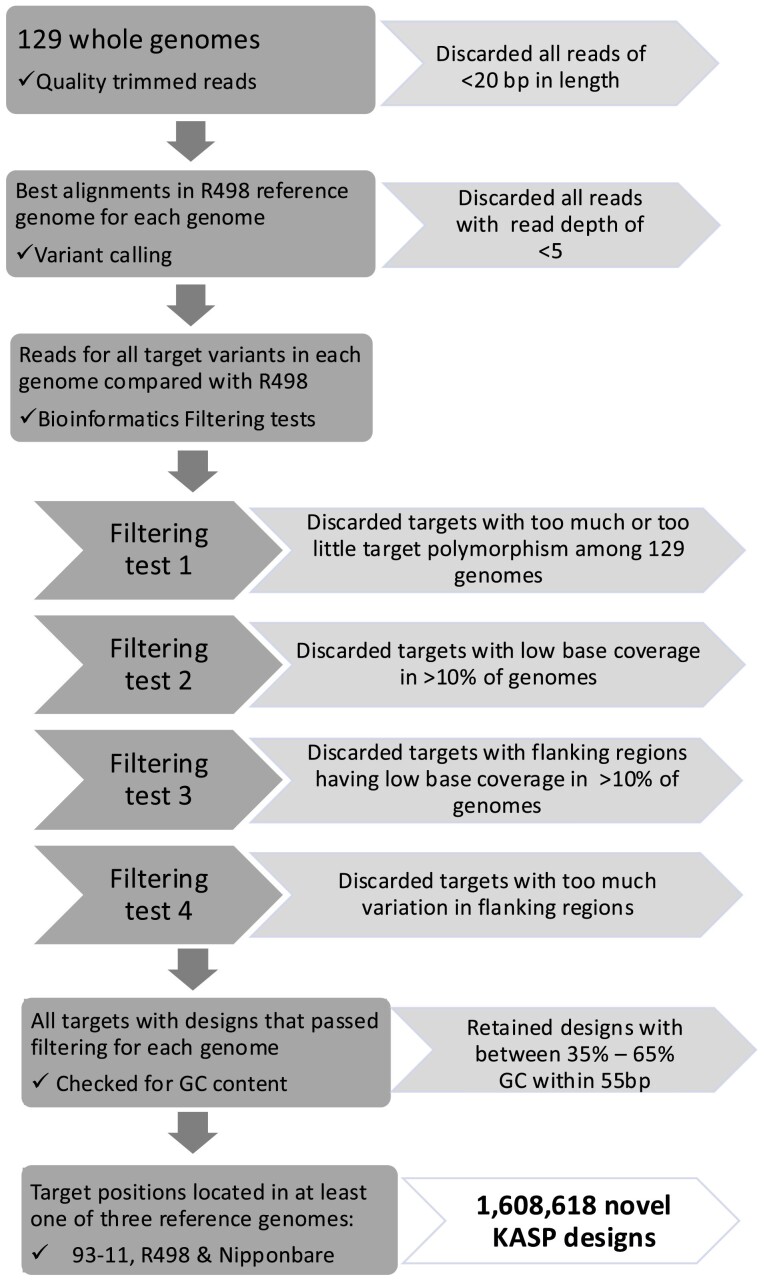
Steps used for incorporation of rice diversity during in-silico design of KASP markers that should discriminate between a R498 reference allele and the most common alternate allele in a population of 129 diverse genomes.

The KASP design and bioinformatics filtering steps were done at Bangor University (BU). In total 78 *indica* genomes and 51 non-*indica* genomes were used for in silico KASP design (Fig. 1). This project incorporated variation from the sequencing data from 118 rice genomes selected and retrieved from the 3,000 Genomes Project (3K RGP, 2014) alongside the paired-end sequencing reads for 11 varieties selected by BU's project partners [9 *indica* rice genomes selected by breeders in Nepal (Steele *et al.* 2018) and 2 Indian upland varieties (Kalinga III and Ashoka 200F)]. Supplementary File 1 contains the methods used for sequencing these 2 previously unpublished genomes. The 118 genomes included at least one line from each of the 89 countries of origin in the 3 K project and all 7 rice varietal groups represented in the 3 K RGP dataset. All genome sequences are available in the EBI Sequence Read Archive, accession numbers PRJNA395505 (for BU genomes) and PRJEB6180 (for 3 K RGP genomes). Tables A and B in Supplementary File S2 provide further details for all genomes used in this study.

### Sequence read processing, alignment, and variant calling

Quality trimming of sequencing reads was carried out using Sickle (Joshi and Fass 2011). An average Phred score of 30 was set as the threshold for the trimming window with sequences truncated at the position of the first N. Trimmed reads shorter than 20 bp were discarded.

Trimmed reads were aligned against the Shuhui 498 (R498) *indica* rice reference genome (Du *et al.* 2017). Version 2 of the genome sequence was downloaded from MBKbase (http://mbkbase.org/R498/) and this version was used for all subsequent R498 alignments and positions generated in this study. Sequence read alignment was carried out with Bowtie2 (Langmead and Salzberg 2012). Alignments were only reported if both mates of a read pair aligned in the expected orientation. A single best alignment was reported for each pair, selected at random in the case of equally good alternative alignments. All other alignment, scoring, and reporting options for Bowtie2 were kept as default.

Genotype likelihood calculation and variant calling was carried out with SAMtools (Li *et al*. 2009). SNPs or insertions with a read depth of <5 were filtered out. Base coverage was calculated using BEDTools (Quinlan 2014).

### Bioinformatics filtering for KASP marker design generation

KASP marker design was carried out utilizing the data from 129 rice genomes using custom Perl scripts through a process of sequential filtering. For each SNP or InDel variation identified in one of the resequenced rice lines, the following tests were carried out to determine whether the variation was suitable for KASP marker design. Here the term “target variation” is used to describe an allele detected in a particular rice line that is different to the allele in the R498 reference genome and under consideration for KASP marker design, and “alternative variation” for any other alternative alleles (up to 2 are possible) at the same genomic site detected in the other rice lines.

1. Removal of rare alleles: If an alternative variation (i.e. alleles other than the R498 allele or the most common target variant at that position) was identified in more than 10% of lines, then the target variation was removed. That is, at least 90% of lines had to possess either the reference allele or the most common alternative allele. In the case of multiple variations fulfilling this criterium for any site, only the most common variant was carried forward. Any target variations not demonstrating polymorphism among the resequenced lines, were also discarded (i.e. where all 129 test lines shared an allele that was different from the reference allele).
2. Removal of targets with low base coverage: In the case of the target variation being a SNP or In/Del the base coverage was checked at the target site. The resequenced genomes utilized variable read depths, and a particular genomic site was considered to have low base coverage if the read depth was <0.1 of the average read depth for the genome in question. If the target variation site was identified as having low base coverage in more than 10% of the 129 resequenced genomes it was not included.
3. Removal of targets with low base coverage in flanking sequences: Tests were then carried out on the 50 bp either side of the target variation, described here as the “flanking sequence” with each base referred to as a “flanking site.” The same base coverage check described in check 2 above for each target site at a SNP or insertion was made for each base position in the flanking sequence, with the target variation being rejected in the case of a single failure at any base position along the flanking sequence.
4. Removal of targets with high variation in flanking sequences: For each base position in the flanking sequence, all 129 genomes were checked for the presence of variations. If alternative variations were present at a flanking site then the target variation was rejected unless: (ⅰ) all the alternative variations in the flanking sequence were insertions or deletions of equal length, there was only one insertion or deletion, no insertion or deletion was within 5 bp of the target variation, and no more than 10 bases were inserted or deleted; (ⅱ) all the alternative variations were at a single base position, i.e. were SNPs, and no more than 5 flanking sites were SNPs.

A KASP design sequence consists of the target variation with the 50 bp flanking either side of it. Preliminary KASP designs were generated for all target variations that had passed the filtering tests 1–4 (above), with degenerate nucleotides included for flanking sequence variants identified among the 129 genomes. SNPs in the flanking sequence were represented using the appropriate International Union of Pure and Applied Chemistry (IUPAC) nucleotide code, and insertions and deletions were represented by sequences of Ns (e.g. NNN for a 3 base deletion or insertion).

The KASP design sequences were checked for the presence of repeats by first removing any Ns from the design sequence and then creating a set of test sequences that represented all the possible combinations of SNPs in the design sequence. If a tandem repeat consisting of more than 5 copies of any 1 to 5 nucleotide patterns was detected in any member of the test set, the target variation was excluded.

To enable end-users to cross-reference marker positions between both *indica* and *japonica* reference genomes, potential KASP design sequences were aligned against the *indica* rice R498 (version 2, Du *et al.* 2017) reference genome and the *japonica* rice Nipponbare reference genome (version IRGSP-1.0, International Rice Genome Sequencing Project 2005; downloaded from https://plants.ensembl.org this version was used for all subsequent Nipponbare alignments and positions generated in this study) using BLAST (Camacho *et al.* 2009). Only design sequences that had a single best alignment in both reference genomes were kept.

A final check for inclusion was for the GC content within a 55 bp window of the KASP design sequence to be between 35% and 65%, any that did not meet this criterion were excluded to optimize their reliability in PCR.

### Cross referencing with the historic *indica* reference genome

The KASP marker design sequences derived via the above filtering steps were aligned against the older *indica* rice 93–11 reference genome assembly (ASM465v1, Yu *et al.* 2002: this version was used for all subsequent 93–11 alignments and positions generated in this study) using BLAST (Camacho *et al.* 2009). Any best-hit sequences with <90% identity over the full sequence length, or those having multiple best hits, were given an unknown position in the 93–11 reference genome. This was done to enable subsequent annotation of gene features, C6IAR SNPs and SSRs to include data from 2 *indica* reference genomes.

### Annotating target variation with predicted function

The gene feature annotations for the same 3 genomes were downloaded in GFF format: The R498 genome (version2, Du *et al.* 2017), Nipponbare [November 2018 release of RAP-DB (Sakai *et al.* 2013)], and 93–11 [2010 release of the BGI RISe Rice Information System (Zhao *et al.* 2004)]. These files were processed with custom Perl scripts to categorize each target variation with respect to location within gene features and record information about predicted effect (e.g. functional/nonfunctional) for each reference genome.

Target variations (SNPs or InDels) were classified as either being intergenic, or genic. Genic variations located within protein coding genes were further classified according to their location in the 5′/3′ UTR regions, introns, or coding sequences. SNPs within coding regions were further categorized according to their predicted effect on the translated amino acid sequence: synonymous, nonsynonymous, premature stop codon, and stop codon loss. Insertions or deletions overlapping coding regions were classified as either frameshift or nonframeshift and start/stop codon loss mutations were identified. The effect on all isoforms was predicted for any variants located within coding genes with multiple annotated transcript isoforms.

### Determination of C6IAR SNP genomic positions

This was done so that database users can cross reference KASP designs with SNPs in the Cornell 6 K Infinium rice array (C6IAR) (Thomson *et al*. 2017). C6IAR SNPs were aligned in the same 3 reference genomes using the same criteria described above for annotating target variation with predicted function. Of the 5,274 C6IAR SNPs, 94% aligned with a position in at least 1 reference genome, with 75% aligning to the same chromosome in all 3 genomes (Table C in Supplementary File S2). Of the 4,569 (86%) C6IAR SNPs that aligned to R498 autosomes only 2,099 (46% of these) fulfilled KASP design criteria (Table D in Supplementary File S2).

### Determination of SSR markers genomic positions

This was done so that database users can cross-reference between previously published SSR markers and the KASP designs in this study. Forward and reverse pairs of primer sequences for 19,475 SSR rice markers were downloaded from www.gramene.org and each primer was aligned using BLAST (Camacho *et al.* 2009) against the R498 (Du *et al.* 2017), Nipponbare (International Rice Genome Sequencing Project 2005), and 93–11 (Yu *et al.* 2002) reference genome sequences.

Individual primer alignments were rejected if they had an identity of <95% for full sequence length. In the case of multiple best hits for SSR primers, all combinations of primer pair alignments were considered. Ninety-eight percent (19,138) of SSR primer pairs used for the analysis fulfilled the criteria for alignment, of which 16,980 (89% of all SSRs considered) aligned with a position in at least one of the 3 reference genomes (Shuhui 498, 93–11 or Nipponbare). Seventy-three percent of aligned SSRs were positioned on the same chromosome in all 3 genomes. Then SSRs were given a known position if both left and right primers aligned within 10 kb of each other and when only a single pair of best hit alignments fulfilled these criteria in at least one reference genome (Table E in Supplementary File S2).

### Selection of 4,000 KASP for validation test and population analysis

The genomic positions of 1,080 breeder-specified target genes or SSR markers previously associated with traits or QTLs were used to identify KASP designs that were situated within 0–19,913 bp of a target gene or SSR position (Table F in Supplementary File S2, where columns B and C, headed “Marker/gene” and “Alternative IDs” give the names or codes used in previous publications or databases for target genes or markers). When more than 5 KASP designs were located within this range, 5 were selected from them according to predicted functionality, followed by closeness to the target. One-hundred and forty-three targets had fewer than 5 KASP designs, and for these all targets were included (Table G in Supplementary File S2). Designs for KASP targeting variations predicted to result in functional mutations were preferentially selected, while designs in very close proximity to others in the set were preferentially removed. This resulted in 5,028 KASP designs selected for their proximity to genes or SSRs commonly targeted in rice breeding programs. This set only included designs that had not been selected for validation in breeding applications being done in the wider project. These designs (the target sequences including ± 50 bp flanking either side) were submitted to LGC who tested them in silico with their proprietary Kraken software for primer design and they rejected 43 designs because they did not pass the criteria for primer production.

From the remaining 4,985 KASP designs (targeting >1,024 loci), 4,000 were selected by: (i) removing all that were within 100 bp of another marker with the same predicted genotype for all varieties; (ii) removing any nonfunctional markers furthest from its SSR target, starting with the targets that have the most markers and continuing until 4,000 remained. These 4,000 designs (for 1,024 loci) were submitted to LGC for synthesis of the corresponding KASP primers for use in genotyping in the rice collection.

There was no selection for C6IAR loci during selection of the 4,000 designs and only 20 of these submitted designs had C6IAR equivalents. There were 3,275 KASP designs selected and submitted in the vicinity of 635 Cornell SSR markers (maximum of 5 designs per SSR).

### Development of a diverse rice panel for genotyping

Diverse rice (*O. sativa*) genotypes, selected to include a wide range of landraces, modern varieties and advanced breeding lines, were supplied by breeders or researchers from: the International Rice Research Institute (IRRI), the National Institute for Biotechnology and Genetic Engineering (NIBGE), Pakistan, the Sheri-e-Kashmir University of Agricultural Sciences and Technology of Kashmir, India (SKUAST), the Nepal Agriculture Research Council (NARC), Anamolbiu PVT, Nepal and the Earlham Institute, UK. Modern varieties or advanced breeding lines (including some for direct seeding in uplands) from Brazil, Bangladesh and Pakistan were sourced from the International Rice Genebank at IRRI. The collection (Table H in Supplementary File S2) included samples originating from 16 countries and 8 breeding programs and their designations included 132 *indica*, 22 *japonica*, 5 boro, and 17 basmati (Table I in Supplementary File S2). Seed samples were grown at BU's Henfaes Research Centre, sown either in May 2018 or June 2019. Seeds were sown directly into compost and grown under the glasshouse conditions described in Note B in Supplementary File S1.

Leaf samples for DNA extraction were taken after about 7 weeks growth from a single plant of each line using BioArk plant sampling kits, with 96 sampled in July 2018 and a further 82 sampled in August 2019. The 178 rice DNA samples were genotyped with KASP by LGC Biosearch Technologies, Hoddesdon, UK.

Genotype data were converted to a numeric matrix (1 = R498 allele; 0 = target variant; heterozygotes were run either coded as the most common allele or as 0.5, and results did not vary) and used for Hierarchical cluster analyses with the FactoMineR and Factoextra libraries in R (Lé *et al*. 2008). Distances were calculated using the “dist” function and the “euclidean” method to give a distance matrix. Clusters were produced from the distance matrix using the method “average” in the function “hclust” and plotted using the function “plot.”

The Wilcoxon rank-sum test was used to test whether various KASP design properties differed between the designs that produced successful genotyping assays among the 178 rice samples and those that did not. The tests related to the number and location of ambiguous bases representing nontarget variations, the number and location of InDel bases (only for KASP designs targeting InDels), and the GC content of the design sequence. The 2 flanking sequences of KASP designs could include either no ambiguous bases or one or more, up to a maximum of 5 (according to the filtering step 4 above). The distances in bp between the target SNP or InDel and the furthest ambiguous base in either or both flanking sequences were used to test properties relating to the distance to the *n*th ambiguous base (where *n* was in the range 0–5). Separate tests were done for left flank distance, right flank distance, shortest distance in either flank and longest distance in either flank. Only designs with *n* or more ambiguous bases in the design the flanking the target sequence was included in the tests relating to the distance between the design target and the *n*th ambiguous base. Only designs with at least *n* ambiguous bases in both flanks were included for tests on properties relating to the longest distance to the *n*th ambiguous base.

### Database development

A “back-end” database was constructed at BU to act as a repository for the KASP assays designed in this study. It contains the bp location of the target variant for each KASP design in up to 3 reference genomes (Shuhui 498, 93–11 or Nipponbare) and the expected variant genotype at each KASP design for each of the 129 source data genomes. Options were included to enable breeders to search for KASP designs, either within a specified region of the genome or at a specified distance from either a named gene, SSR or a SNP from the C6IAR panel. Each KASP design from this study was assigned a KASP ID number (pKey) for information management. This back-end database was provided to LGC for them to use to update their Rice Assay Search Tool. LGC released a beta version of the search tool which has been tested by the authors and 2 independent rice breeders. A user's manual was written by BU and LGC (Supplementary File S3).

## Results

### Sequence read alignment to *indica* reference genome

Rates of alignment for 129 rice whole genome sequences with the R498 *indica* reference genome (Du *et al.* 2017) ranged from 52.4% to 95.4%. Genome coverage was between 81.9 and 97.4% and average read depth ranged from 6.2 to 90.3 (Table B in Supplementary File S2). Across all 129 genomes, a total of 15,140,996 variant sites were identified, with an average of one variant site for every 25.8 bases in the 391 Mb R498 genome. The number of variant sites for each variety against R498 ranged from 737,046 to 2,343,000.

### Novel KASP marker designs

**Fig. 2.**
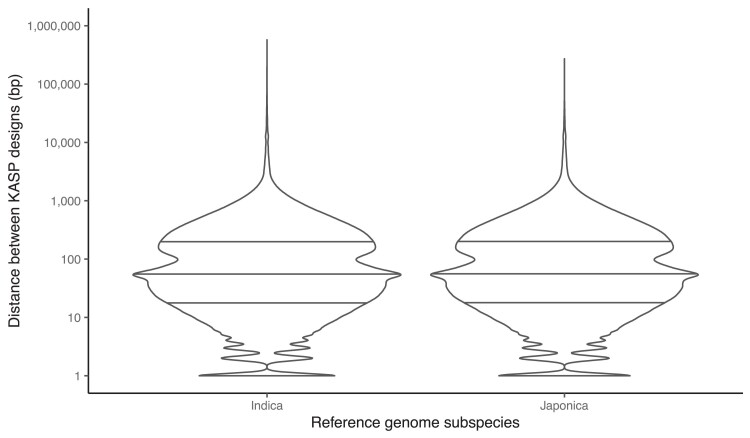
Distribution of distances between adjacent KASP designs aligning in the Shuhui 498 *indica* (Du *et al.* 2017) and Nipponbare *japonica* (International Rice Genome Sequencing Project 2005) reference genomes. Horizontal lines represent the first quartile, median, and third quartile.

**Fig. 3.**
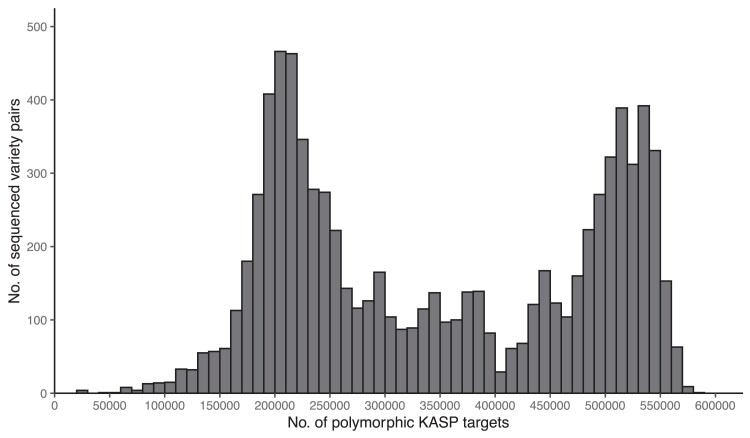
Number of polymorphic sites identified in the 8,256 possible pairwise crosses of the 129 diverse sequenced rice lines used for KASP design generation.

Over 1.6 million KASP marker designs were generated in silico by this bioinformatics study. Based on their positions in relation to the gene models of the *japonica* Nipponbare and *indica* R498 genomes, between ∼76,000 and 98,000 design targets were predicted to be functional variations that would cause a change in the expressed proteins (Table 1). The maximum distance between adjacent KASP marker designs was 573 kb for the *indica* reference genome and 270 kb for *japonica*, with the median distance between designs for both being 55 bp (Fig. 2). The number of KASP targets predicted to be polymorphic between all 8,256 pairs of possible varietal comparisons ranged between 23,078 (minimum) and 581,415 (maximum), with a median of 328,256 (Fig. 3).

**Table 1.** Total number and number of predicted functional KASP designs generated per chromosome.

Chromosome	*Indica* (Shuhui 498)		*Japonica* (Nipponbare)	
	Total	Functional	Total	Functional
1	190,895	12,780	191,156	10,252
2	166,771	10,565	166,949	8,134
3	171,281	9,696	170,739	7,128
4	130,900	8,752	130,880	6,573
5	141,145	7,210	141,455	5,284
6	144,269	8,444	144,479	6,573
7	130,922	7,731	130,778	6,122
8	122,189	7,147	122,194	5,613
9	96,906	5,951	96,910	4,491
10	100,483	5,524	99,552	4,355
11	116,812	8,119	116,167	6,359
12	96,040	6,319	95,971	4,830
Chloroplast	0	0	n/a	n/a
Mitochondrion	5	0	n/a	n/a
Unanchored contigs	n/a	n/a	1,388	0
Total	1,608,618	98,238	1,608,618	75,714

### Demonstration of utility of novel KASP panel through genotyping

**Fig. 4.**
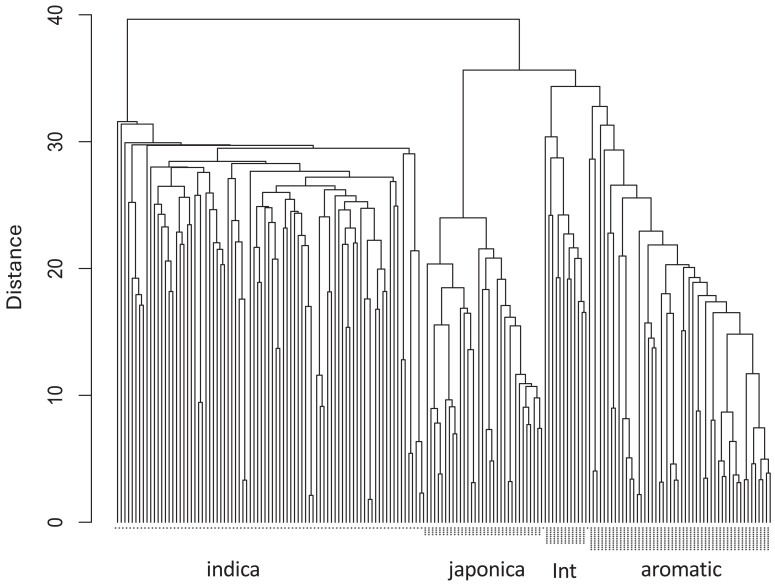
Hierarchical cluster analysis of 178 genotypes with the 3,355 polymorphic KASP markers. The groups from the PCA are indicated on the y axis (* indica group, *** japonica group, ***** intermediate group (Int), ******* aromatic group).

Of the 4,000 trait-specific KASP designs tested, 3,371 were passed for wet-lab validation according to the service provider. But more stringent data analysis revealed that 3,366 KASP gave successful genotype calls in >90% of samples, which resulted in successful KASP (Tables I and J in Supplementary File S2); hence, 3,366 were considered as validated. Eleven markers were monomorphic in this set of rice germplasm so data for the remaining 3,355 markers were used in cluster analysis to reveal separation into 4 major groups corresponding to *indica*, *japonica*, intermediate, and aromatic subtypes. This grouping well-reflects the diverse population tested which includes diverse landraces as well as breeding lines and modern varieties, many of which are derived from crosses between subtypes (Fig. 4).

Some 635 designs (15.9%) failed in all samples (Figure Aa in Supplementary File S1) and for designs with genotype calls below 90%, the lowest call rate was 17.4%. For the successful 3,366 markers, 23.5% produced calls in all samples while 81.9% produced calls in >90% of samples (Figure Ab in Supplementary File S1). The percentage successful allele calls per variety ranged from 72.5% to 83.8%. There was no significant difference in success rate of KASP between different rice subgroups or countries of origin.

### Comparison between sequenced and genotyped datasets

Thirteen of the genotyped rice samples had the same names as 13 of the 129 sequenced genomes used in the marker design process. For each of these named lines the genotype calls for each marker were compared against the sequenced genotypes (Table L in Supplementary File S2). There were 3,389 nonmatching calls (7.7%) out of 43,758 datapoints in this subset. Included in nonmatching calls are failed calls, which were reported in the dataset as either “Bad,” “Uncallable,” and “?”. In the subset of 13 varieties there were 3 Bad calls (1 each in Anmol Masuli, Lok Tantra, and Kalinga III at different loci); 2 Uncallable (in Loc Tantra and Kalinga III at different loci); and 501 calls of “?”, ranging from 20 to 63 alleles called as “?” per line.

The percentage matching alleles for line ranged from 97% to as low as 46.5%. However, >77% of markers had calls matching predicted genotypes in all but one line (Chommrong) and 10 of the 13 lines had >88% agreement between the genotype data and sequence data.

There was complete agreement for 1,043 (31%) markers and nonagreement for the remaining 69%. Of these, 1,610 (48%) did not match in only 1 line. The number of nonagreements reduced rapidly for additional lines: 14% in 2 lines for, 5% in 3 lines and 0.9% in 4 lines. Only 19 lines had nonmatching calls in 5 or more lines. Only 1 marker (R498 locus 10:20,882,568) had no matches in all 13 lines, but all were called as heterozygotes (possibly an artifact, see Discussion).

Success rates for the 13 lines used in design generation were compared against the 165 that were not. They had median success rates of 83.33% and 83.05%, respectively, and a Wilcoxon rank-sum test showed no significant difference in the distributions of success rates at the 99% confidence level, with a *P*-value of 0.035.

### Effect of ambiguous bases in designs on success rate

Examining the subsets of the 4,000 KASP designs submitted for genotyping showed that KASP designs with a mean of 2.5 ambiguous bases were significantly more likely to fail than those with a mean of 1.86 ambiguous bases (Table 2). Several properties of the distance of ambiguous bases from the target variation also showed significant differences in distribution between the successful and failed markers (Table 2). KASP designs with higher GC had significantly more failures. Wilcoxon rank-sum test results for all 29 properties are shown in Table 2.

**Table 2.** Results of Wilcoxon rank-sum tests (*W*) of difference between properties of KASP designs resulting in successful and failing assays.

KASP design property	*W*	*P*-value	Mean	
			Success	Failure
No. of indel bases	2,187	0.689	3.67	3.38
Distance of indel from target variation	2,285	0.984	31.24	31.62
No. of ambiguous bases	1,327,001	1.20 × 10^−19^***	1.86	2.5
No. of ambiguous bases in left flank	1,243,608.5	7.19 × 10^−10^***	0.92	1.21
No. of ambiguous bases in right flank	1,263,164	5.09 × 10^−12^***	0.94	1.29
No. of ambiguous bases in flank with least	1,242,962.5	1.48 × 10^−11^***	0.43	0.62
No. of ambiguous bases in flank with most	1,317,236	1.01 × 10^−18^***	1.43	1.87
Distance to first ambiguous base in left flank	371,206.5	0.075	20.22	18.58
Distance to first ambiguous base in right flank	372,326.5	4.9 × 10^−5^***	20.32	17.34
Shortest distance to first ambiguous base in either flank	364,947	3.63 × 10^−6^***	15.44	12.32
Longest distance to first ambiguous base in either flank	164,961.5	7.39 × 10^−4^ ***	27.98	25.26
Distance to second ambiguous base in left flank	90,414	0.365	29.61	28.84
Distance to second ambiguous base in right flank	93,176	0.004**	29.98	27.41
Shortest distance to second ambiguous base in either flank	92,249	0.002**	27.65	24.94
Longest distance to second ambiguous base in either flank	9470	0.014*	39.4	36.9
Distance to third ambiguous base in left flank	16,438	0.937	34.91	34.87
Distance to third ambiguous base in right flank	13,871.5	0.082	35.34	33.28
Shortest distance to third ambiguous base in either flank	13,879	0.083	35.28	33.16
Distance to fourth ambiguous base in left flank	741.5	0.007**	39.52	34.81
Distance to fourth ambiguous base in right flank	1173.5	0.844	38.77	37.86
Shortest distance to fourth ambiguous base in either flank	1152	0.734	38.77	37.59
Distance to fifth ambiguous base in left flank	12.5	0.435	40.67	45
Distance to fifth ambiguous base in right flank	39	0.456	38	42
Shortest distance to fifth ambiguous base in either flank	39	0.456	38	42
%GC content	1,192,550	9.40 × 10^−5^***	44.07	45.82
Left flank %GC content	1,210,513	4.74 × 10^−6^***	44.44	46.4
Right flank %GC content	1,165,491	3.71 × 10^−3^***	43.61	45.19
Lowest flank %GC content	1,184,005	3.30 × 10^−4^***	40.07	41.56
Highest flank %GC content	1,197,391	4.35 × 10^−5^***	44.07	45.82

*P values <0.05, **P values < 0.01, and ***P values <0.001 (at 99% confidence level).

The overall success rates are likely improved by reducing the cutoff for the maximum number of ambiguous bases permitted in a KASP design. However, this comes at a large cost in the reduced number of potential designs and hence an increased distance between adjacent markers (Table 3).

**Table 3.** Effect of reducing the cutoff for the maximum number of bases permitted in the flanking sequences of KASP designs on number of available designs and predicted success rates.

All potential KASP designs			Genotyped KASP markers		
Maximum number of ambiguous bases	No. of designs identified	Median distance between designs (bp)	No. of markers tested	% markers producing genotyping results	% successful assays
4	1,392,359	68	3,542	85.09	83.76
3	1,135,654	93	3,121	86.48	85.16
2	845,121	144	2,527	87.65	86.36
1	532,572	242	1,787	89.20	87.92
0	223,783	580	828	88.53	87.44

Distances between markers are based on their position in the Shuhui 498 *indica* reference genome. Predicted success rates are calculated from the subsets that fulfill the cutoff criteria out of the 4,000 markers submitted for genotyping in 178 rice lines.

### Proximity of validated KASP to widely used Cornell markers

All 20 of the KASP designs with a Cornell C6IAR equivalent included in the 4,000 designs tested were successfully validated (Table C in Supplementary File S2). The success rate of KASP designs located near to Cornell's SSRs was 81%, with 620 unvalidated KASP designs in the vicinity of SSR markers listed on the Gramene database. Breeders can use Table K in Supplementary File S2 to identify selected designs for trait selection that are close to previously published SSR loci.

### Rice assay search tool

Breeders and other end-users can access the database containing details of the ∼1.6 million KASP assay designs developed in this study through the Rice Assay Search Tool (www.biosearchtech.com/kasp-assay-search) (Further details and search tips are provided in Table M in Supplementary Files S2 and S3).

## Discussion

Previous studies have also mined genomic variations within the rice 3K RGP (2014) data to identify a large number of target SNPs for use by rice researchers (Alexandrov *et al*. 2015; Tareke Woldegiorgis *et al*. 2019). Others have identified KASP for specific traits in rice (e.g. Addison *et al*. 2020; Angira *et al*. 2019; Sandhu *et al*. 2022). To our knowledge, no large-scale previous study has designed KASP primers which include ambiguous flanking variation, or specifically selected thousands of KASP for loci that are relevant to breeding programs.

This study used a bioinformatics pipeline to filter ∼15 billion potential target variants detected among 129 publicly accessible rice genomes and remove those within problematic regions as well as any with unsuitable nontarget variations flanking the target variation. It used the R498 *indica* reference genome as the baseline for KASP targets, meaning that every KASP we designed assayed for the R498 allele and the most common alternate allele at that target among a sampled population of 129 resequenced genomes. Targets having multiple alternate alleles where they together accounted for >10% of that population were not developed as KASP in this study.

This pipeline resulted in ∼1.6 million KASP assay designs optimized to include IUPAC nucleotide codes at a maximum of 5 nontarget variations in each region flanking the target SNP or InDel. The number of KASP assays generated compares well with other rice KASP development projects (Cheon *et al*. 2018). Similar numbers of KASP designs were present in both *indica* and *japonica* genomes although *indica* had more potentially functional markers (Table 1) and slightly larger gaps between markers (Table 3).

The frequency of polymorphic sites showed a bi-modal pattern when plotted as a histogram for pairwise comparisons between genotypes (Fig. 3). A similar bimodal pattern of polymorphisms observed by Alexandrov *et al*. (2015) was considered to indicate the absence of a proportion of mapped reads in some genomes. There were differences in coverage between the genomes used in this study. However, it was observed that the number of polymorphisms in pairs was associated with how closely related each pair are to each other, with pairs in the peak on the left side of the histogram made up of varieties from the same *Oryza* subgroup group while those on the right are made up of 2 varieties from different groups (aus, boro, *indica*, *japonica,* etc.). Pairs with intermediate values are the product of lower polymorphic pairs between groups or higher polymorphic pairs within groups.

### Limitations of the design pipeline

The KASP design algorithm used in this study resulted in fewer designs generated (10.6% of the ∼15 million variant sites identified in the sequenced lines) than the 51.9% variation site to KASP design conversion rate reported in our previous study (Steele *et al.* 2018). This was expected because of the wider range of varieties used in the current study giving many more variant sites in the flanking sequences of target SNPs and InDels. The avoidance of potential markers with excessive variation in flanking sequences has reduced the overall number of successful KASP designs for targets that are predicted to be polymorphic at target sites. In practical terms, particular combinations of parental lines may have large genomic regions lacking detectable polymorphism using the available assay designs.

When there is polymorphism in regions harboring specific traits, these markers offer precision and effectiveness for trait selection. However, if breeders' populations do not show polymorphism with any of these markers in specific regions (e.g. for fine mapping) they can consider de novo cross-specific marker design generation (without ambiguous bases), which can be carried out using the KASP design software code provided by Steele *et al*. (2018).

### Factors affecting success of KASP assays

Of the assay designs submitted, 99.2% passed the final in-silico step for primer design. Of the subset of 4,000 KASP designs developed into “wet lab” assays, 84% were successfully amplified with alleles called. This rate was only slightly lower than was obtained for KASP designed from only 9 genomes without the inclusion of ambiguous bases (Steele *et al*. 2018) and for KASP designed from previously published rice SNPs by Yang *et al*. (2019). It is higher than the success rate of 71% validated KASP converted from SNPs derived from RNA-seq in maize (Jagtap *et al*. 2020).

Of the 3,366 validated KASP, 82% gave allele calls in a panel of 178 diverse varieties, providing genotypes for >90% of the panel. No significant differences were observed in the genotyping success rates of 116 not-resequenced lines as compared with 13 genotyped resequenced lines that were used in the assay design. The rates are within the range of other KASP design studies in rice (e.g. 70% reported by Gouda *et al*. 2021). Overall, this result indicates that these KASP assays have a high probability of working in a wide range of rice populations and should be considered widely applicable for breeding. The following discussion considers some of the reasons that could lead to failure for genotyping.

In the 13 varieties used for both KASP design generation and genotyping, the vast majority of genotype calls matched those predicted in the design stage, with the percentage of matching genotypes being within the bounds of normal within-variety variation. The notable exception was Chhomrong for which only 46% of genotype calls matched expectations. Clearly there were genetic differences between the 2 seed lots of Chhomrong used for genotyping in this study and for resequencing the 3K RGP (2014). Chhomrong was originally considered a landrace and subsequent selection and purification produced the released variety with the same name (Joshi *et al*. 2017), which was the source of the sample used here for genotyping. Chhomrong did not have more heterozygote calls than other lines, however the line FL_478 had only 74% agreement with the sequenced version and the disagreement was exacerbated by numerous heterozygous calls in the genotyped sample (Table L in Supplementary File S2).

Nearly 15.8% of the 4,000 marker designs submitted for genotyping failed to result in any genotype calls in this study. Failures might be explained by the extracted DNA quality, the assay conditions in a particular genotyping run or they could potentially be due to issues related to using ambiguous bases in the designs.

From the KASP assay designs submitted for validation, it was possible to infer the aspects most likely to influence success rates. A statistical comparison of various design-related properties suggested that as the number of ambiguous bases increased to accommodate nontarget variations, the rate of success decreased (Table 3). The position of ambiguous bases within the design sequence also affects the chances of success, with a greater distance between target variations and ambiguous bases resulting in a higher proportion of successful designs. The distance between ambiguous and target variation did not show significant differences between successful and unsuccessful marker designs, although there were relatively few designs with a high number of ambiguous bases so statistical power was reduced (Table 2). Reducing the number of permitted ambiguous bases in the KASP designs led to a relatively small increase in assay success rate (Table 3), but the predicted number of potential designs is reduced substantially, and there are larger gaps in genome coverage (Table 3). If no ambiguous bases are permitted in the flanking sequences, then the predicted success rate increases by only ∼5%, but the median distance between markers is increased by more than 10 times, and the number of potential designs is reduced by 86% (Figure B in Supplementary File S1).

The GC content was also linked to design success rate (Table 2). If the resultant primers are leading to failed assays due to nonoptimal assay conditions for the primer GC content, then it is possible that adjusting assay conditions could result in successful genotyping.

No significant differences were observed between the distributions of the number of inserted or deleted bases of passing and failing designs, nor in their distance from the target variation (Table 3). This may be because only a single insertion or deletion was permitted in the design algorithm and, thus, any InDels could be avoided in primer design.

Although genotype calls with question marks (?) were rare in the diverse population (0.011%), it is noteworthy that they often occurred in several different genotypes at the same locus, suggesting they have a biological cause rather than being an artifact. For example, one marker (locus R498 position 2:31326133) had 47 of these calls, 55 homozygous A calls (the alternate allele used in the KASP design) and 76 homozygous T calls (the R498 target allele) in the diverse population (Table J in Supplementary File S2). In contrast, the same marker had no calls for the alternate allele (A) in a different population largely composed of commercial aromatic rice varieties genotyped by Steele *et al*. (2021). In that study, all genotype calls were either “?” or T homozygote. Our working hypothesis is that the “?” calls in both studies may denote presence of an alternate (“third”) allele that was not included in the designs (either in a homozygote or heterozygote state) in the accessions with “?” calls. Further work is needed to test this hypothesis either using sequencing of accessions carrying the “?” allele or by querying the genome assemblies of such accessions. An alternative approach could be to produce alternative KASP assays at these loci with primers selected to call the rarer alternate allele instead of the most common one, which was our default strategy during KASP design in this study. Anecdotally, breeders in Nepal, Anmolbiu PVT and NARC, who used such KASP markers for selective breeding have found that the calls for “?” segregate as expected in some populations, and often can show an identical pattern of segregation to adjacent, tightly linked, markers with clarity in calls, indicating that data from such markers can be used, in some circumstances and with caution, to inform selection decisions.

### Efficacy and value for breeding applications

The novel panel of 3,366 “ambiguous base” trait-specific KASP developed in this study were validated in a panel of diverse rice, demonstrating the efficacy of such designs for genotyping use across a wide range of potential varieties. By sampling multiple KASP for 1,024 target loci, we hope to have widened the range of assays available so that breeders can select the ones that are most useful in their crosses.

Many of the markers located near to specific targets or genes are relevant to multiple breeding programs. Some of the validated trait-specific markers have been functionally confirmed by breeders to be linked to traits including disease resistance genes *Xa5*, *Piz-t*, *Pi33* (Arif M., NIBGE Personal communication) and QTL for bakanae foot rot resistance (Shikari A., SKUAST, Personal communication). Nepalese breeders at NARC and Anamolbiu PVT have used KASP designs from this study in marker-assisted backcrossing to successfully incorporate blast and bacterial leaf blight resistance genes in Khumal-4, Sunaulo Sugandha, Sugandha-1, and Anmol Mansuli that are being tested for potential release in Nepal.

The applied validation of the ∼4 K KASP panel was demonstrated through their ability to resolve groups in hierarchical cluster analysis (Fig. 4). It is noteworthy that members of the intermediate group derived through this analysis were 2 Vietnamese varieties (Khara Ganga and OM 479 expected to be *indica*) and the approved Basmati variety Pusa Basmati 1. Two varieties originally thought to be japonica (SKAU_D40 and SKAU_D54) were confirmed as *indica* in this analysis, supporting a similar finding by Shikari *et al*. (2021) with a different subset of our KASP designs. Those authors successfully used 114 (of 213 genotyped loci) of our marker designs for genotyping and structural analysis of a 470 line population of Himalayan-grown rice. At the same time (in the same LGC project), this subset of markers were also genotyped on the USDA minicore collection and Pakistan landraces and also applied successfully for population structure analysis (M. Arif, NIBGE, Pakistan, Personal communication), supporting the value of the wider set of KASP designs for this application, and also highlighting their potential high-throughput scalability. With coordinated teamwork and careful management of resources, a large subset of KASP can be used efficiently for screening large populations including multiple sets of material from different groups to increase efficiency.

In contrast to SSR markers, which can detect multiple alleles at a single locus, KASP only detect a maximum of 2 alternate alleles (SNPs or InDels) at each target locus (although in some cases a repeatable null allele can be identified that follows expected Mendelian patterns of inheritance, and thus inferred as a “third” allele, discussed above). For genetic diversity studies, estimates suggest that 7–11 times more KASP markers are needed to reveal a similar amount of diversity (in the form of haplotypes) compared with a single SSR (Hamblin *et al.* 2007; Van Inghelandt *et al*. 2010).

For breeding applications, Ashfaq *et al*. (2023) used a subset of KASP derived from this study and found that a similar number of foreground or background marker loci are required for KASP as compared with SSRs when applied for QTL mapping and haplotype discovery, so long as KASP markers known to be polymorphic in the population were used. The number of assays can be scaled up for high-throughput applications such as genomic selection or down for marker-assisted backcrossing and panels including more as yet unvalidated KASP can be selected from the online rice assay search tool. The rice assay search tool links each marker to genome annotation information and contains information about predicted gene functionality as well as alleles in resequenced genomes. The resources in the search tool could be used by researchers to integrate these KASP with other Omics data.

## Supplementary Material

jkae251_Supplementary_Data

## Data Availability

The genome sequences used for KASP design development are publicly available via the EBI Sequence Read Archive, accession numbers PRJNA395505 for BU genomes (www.ebi.ac.uk/ena/browser/view/PRJNA395505) and PRJEB6180 for 3 K RGP genomes (www.ebi.ac.uk/ena/browser/view/PRJEB6180). Genomic locations of all validated KASP are available in supplemental files. Genomic locations of KASP target variants for all ∼1.6 M KASP designs generated during this study are available via the BU-LGC_plus rice assay search tool: www.biosearchtech.com/kasp-assay-search. Supplemental material available at G3 online.
