# Supplementary material for: Thousands of trait-specific KASP markers designed for diverse breeding applications in rice (Oryza sativa)
Source: G3 (Bethesda). 2024 Nov 1;15(1):jkae251. doi: 10.1093/g3journal/jkae251 (PMC11708223; doi:10.1093/g3journal/jkae251)
Supplement: jkae251_Supplementary_Data [file jkae251_supplementary_data.zip › File_S1_G3-2024-405461.pdf]

Steele K.A., Quinton-Tulloch M., Vyas D. & Witcombe J.R., 2024, G3: Genes|Genomes|Genetics

Supplementary File S1
Note A
Methods for sequencing two previously unpublished genomes

Methods for growing and sequencing Ashoka 200F and Kalinga III

Two Indian upland rice varieties were resequenced for this study: Kalinga III (bred by the Central Rice Research Institute (CRRI), Cuttack, and released in 1983 for rainfed upland rice ecosystems in eastern India) and Ashoka 200F (released in 2001 as Birsa Gramin Dhan 109; selected from a bulk- population derived from a cross between Kalinga III and IR64 as described by Virk et al., 2003). Seeds of the two varieties were obtained from Birsa Agricultural University in 1999 and maintained as pure line cultures at Bangor University. Seedlings were grown for 1 month in John Innes No. 2 compost with supplementary lighting for 8 hours daily (minimum photon flux density of photosynthetically active radiation, 400–700 nm, of 350 mmol m^{-2} s^{-1}) in a greenhouse at Bangor, North Wales, at 25 -28°C day and 20°C night temperatures. DNA was extracted from 100 mg leaf material from a single plant of each variety using a Qiagen DNA Plant kit (Qiagen, UK), following the manufacturer's instructions. LGC Biosearch Technologies (Berlin, Germany) carried out 150 bp paired-end read sequencing on an Illumina NextSeq 500 V2 platform.

Supplementary File S1
Figure A

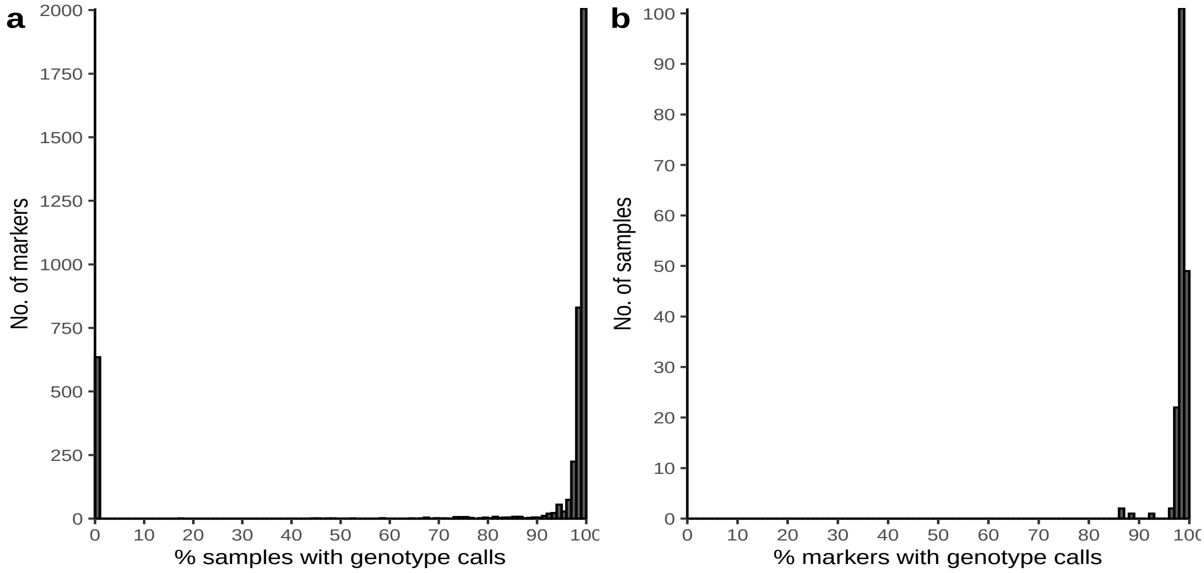

Figure A. Success rates from genotyping of 178 samples with 4,000 KASP markers (a) Percentage of samples with genotype calls for each marker; (b) Percentage of markers with genotype calls for each sample.

Supplementary File S1

Note B

Notes on naming of KASP markers tested for validation during research by BU/LGC+project partners

We named 5000 of the designs for testing in this study on 178 genotypes using the following format:
<target_id>_<variation_type>_<functional_status>_<marker_no>

The functional status part of the design ID is a two-letter code representing whether or not the variation is predicted to be a functional mutation (f) or not (n) according to the indica and japonica genome annotations (e.g. ff = predicted functional mutation in both; nf = predicted non-functional in indica, functional in japonica).

So, for example, the ID 'RM17496_SNP_ff_1' corresponds to a KASP design selected due to its proximity to the RM17496 marker, is based on a SNP, and is predicted to be functional mutation in both subtypes.

We named 1968 designs for testing on breeders lines[1,2] with the format:
gs_Id#
Where # 1-1968 corresponds to the marker number in chromosome position order.

Other formats:
Some KASP names were assigned to correspond with a target gene or SSR in published information such as the Grameme. This format was used of the designs validated for Basmati analysis[3].

References

1. Shikari et al. (2020) KASP™ based markers reveal a population sub-structure in temperate rice (*Oryza sativa* L.) germplasm and local landraces grown in the Kashmir valley, north-western Himalayas. Genetic Resources and Crop Evolution 68: 821-834.

2. Ashfaq et al. (2023) KASP mapping of QTLs for yield components using a RIL population in Basmati rice (*Oryza sativa* L.) Euphytica 219:79

3. Steele et al. (2020) Developing KASP Markers for Identification of Basmati Rice Varieties. Food Analytical Methods 14: 663-673

Supplementary File S1
Figure B

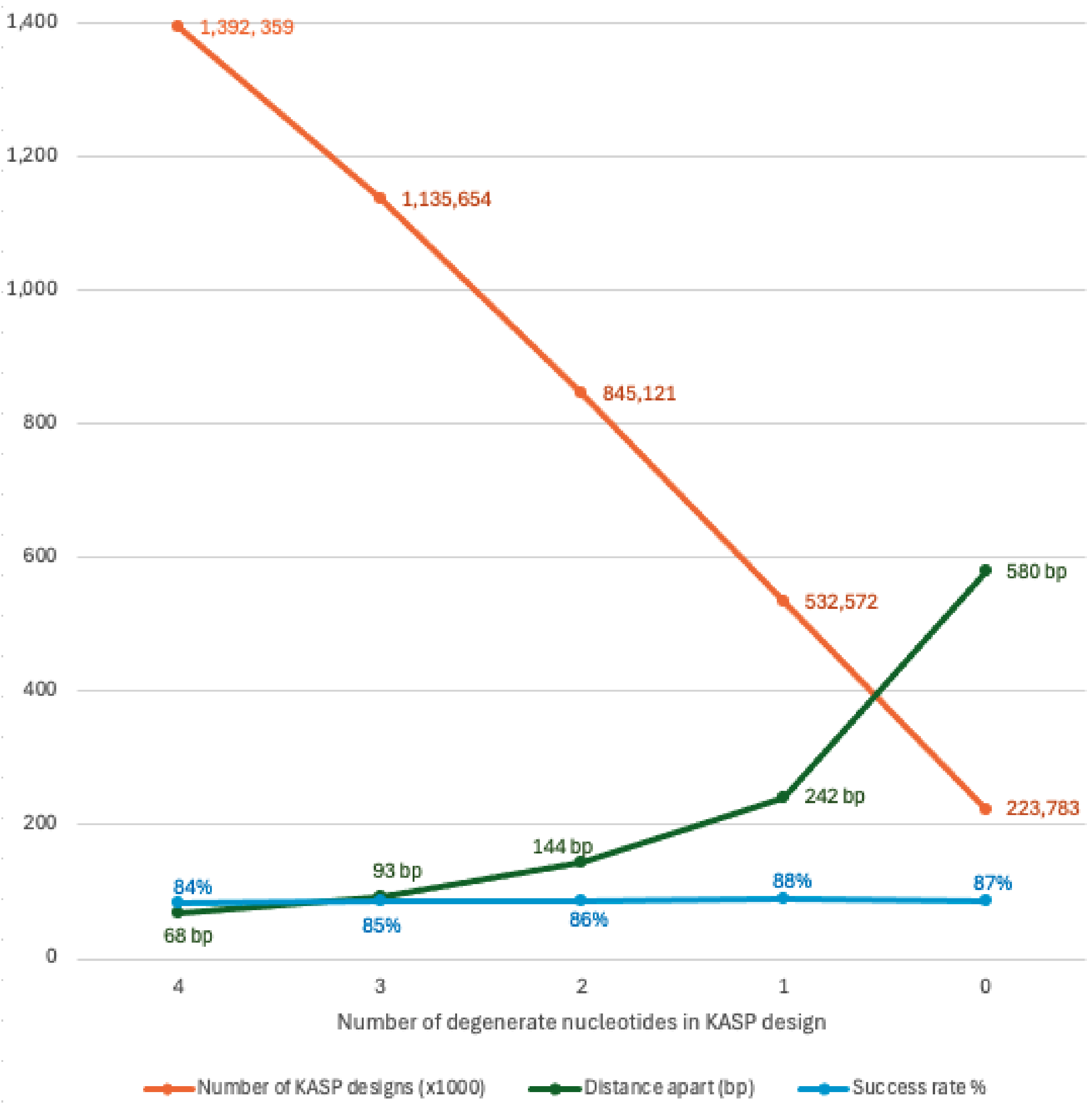

Figure B. Relationship between number of degenerate nucleotides in KASP design and the number of possible designs (predicted), distance between KASP designs (predicted)and percentage of successful assays (actual). Data used for this plot are presented in Table 3.
