# Supplementary material for: Thousands of trait-specific KASP markers designed for diverse breeding applications in rice (Oryza sativa)
Source: G3 (Bethesda). 2024 Nov 1;15(1):jkae251. doi: 10.1093/g3journal/jkae251 (PMC11708223; doi:10.1093/g3journal/jkae251)
Supplement: jkae251_Supplementary_Data [file jkae251_supplementary_data.zip › File_S3_G3-2024-405461.pdf]

Steele K.A., Quinton-Tulloch M., Vyas D. & Witcombe J.R., 2024, G3: Genes|Genomes|Genetics

BU_LGC_plus Rice genotyping panel

The markers established in the BU_LGC_plus rice panel were developed as a partnership between Bangor university, LGC Biosearch Technologies, Anamolbiu Pvt Ltd (APL), Nepal Agricultural Research Council (NARC), National Institute for Biotechnology and Genetic Engineering (NIBGE), Sher-e-Kashmir University of Agricultural Sciences and Technology (SKUAST), with funding from Innovate UK.

In the 2021/2022 crop year, about 509.87 million metric tons of rice was consumed worldwide[1], making rice one of the top three staples consumed globally. To ensure that new rice variety development can continue uninhibited by future challenges, this communally accessible database of KASP markers was established through the partnership.

The database distinguishes more than 1.3 million potential KASP markers[2] (including >90,000 functional markers). The KASP designs have incorporated the diversity contained within 129 rice genomes representing varieties sourced from 89 countries.

Some of the KASP markers are in proximity to some established SSRs and gene IDs (C6AIR and rapdb) frequently used in marker assisted selection strategies.

Of the designs, 4950 KASP markers were wet-lab validated on a diverse selection of both *Indica* and *Japonica* rice varieties.

Genomic locations in this Rice Assay Search database correspond to the following *Oryza sativa* reference genomes:

- **Shuhui498** (*indica)* assembly project and annotations (Version 2) (Du et al. 2017. Sequencing and de novo assembly of a near complete indica rice genome. Nat Commun 8, 15324).
- **Nipponbare** (*japonica*) genome assembly IRGSP-1.0 (International Rice Genome Sequencing Project, 2005; downloaded from https://plants.ensembl.org) and annotated gene models imported from RAP-DB, November 2018 release (Zhao et al. 2004. BGI-RIS: an integrated information resource and comparative analysis workbench for rice genomics, Nucleic Acids Research, 32,1: D377–D382)
- **93-11** (*indica*) genome assembly (ASM465v1) and annotation of Beijing Genome Institute annotation (Yu et al. 2002. A Draft Sequence of the Rice Genome (*Oryza sativa* L. ssp. *indica*) Science 296,5565: 79-92.)

C6AIR SNPs are from the following publication: Thomson et al. Large‐scale deployment of a rice 6 K SNP array for genetics and breeding applications, Rice (N Y). 2017 Aug 30;10(1):40. doi: 10.1186/s12284-017-0181-2.

Gene ID database searches refer to ontology criteria set out in the Rice Annotation Project database (rapdb.dna.affrc.go.jp)

References

1. Total rice consumption worldwide from 2008/2009 to 2021/2022 (in 1,000 metric tons) (01/02/2022). Statista. https://www.statista.com/statistics/255977/total-global-rice-consumption/

2. Accelerating public sector rice breeding with high-density KASP markers derived from whole genome sequencing of indica rice. K. A. Steele et al. Mol Breeding (2018) 38: 38, https://doi.org/10.1007/s11032-018-0777-2.

Assay Search

LGC Biosearch Technologies have developed Assay Search tools for helping users interested in the KASP assay panels find the assays corresponding to their specific needs.

Create an account, or login with your existing credentials, and access this unique database to screen across your specific criteria and download the list of resulting KASP markers. The list of results can be downloaded and used to order the corresponding KASP assays.

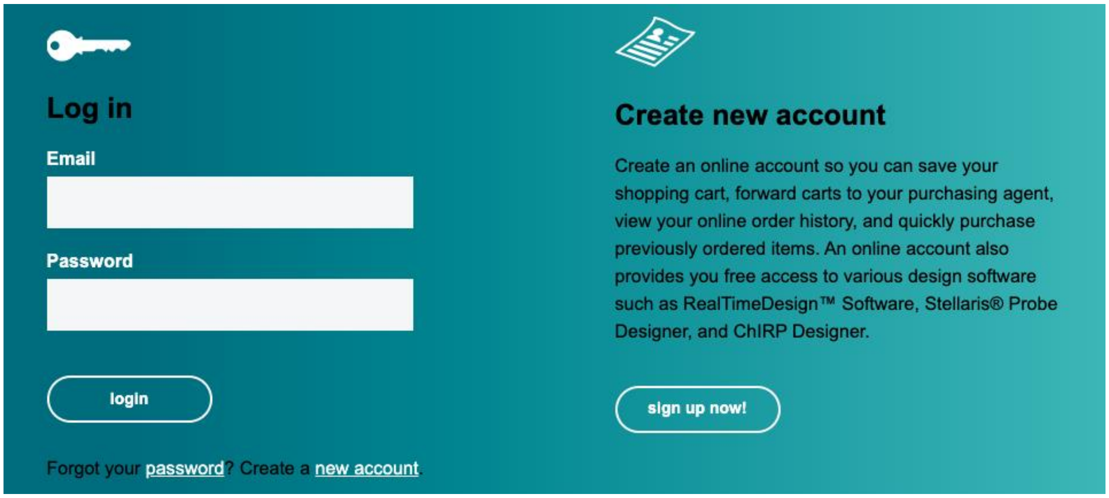

- Search by Gene ID
- Search Gene ID and flanking regions
- Search by microsatellite ID or C6AIR ID
- Search by microsatellite ID or C6AIR ID from position on chromosome
- Search by genome position

Note: Lists of markers are also available in the Resources tab

Search by Gene ID

This search will give KASP marker designs within a specified gene. The gene IDs can be full or partial, eg a search in Nipponbare for OS01G09 would find all gene IDs starting with OS01G09, while OS01G0960800 would find only the requested gene details. Upstream/Downstream regions can also be set to increase the search range further.

Select a specified chromosome and find KASP markers between your chosen start and end positions.

Search tips:

Please be cautious that the search tool is case-sensitive and that the gene IDs used for the search may require different formats depending on the Genome. Also, make sure that you are not including spaces in the text typed. Please carefully read the instructions below.

1. If the gene ID or name gives no hits, refer to the Rice Annotation Project database (rapdb.dna.affrc.go.jp) and use the keyword search to find the RAP gene ID corresponding to the gene name that you want to look up.

2. For searches in Nipponbare (*japonica*) the gene ID should start with OS. Enter the gene ID in the box using only uppercase letters and always use a G as the fifth character and ignore any dash and further digits: e.g for the gene known as Waxy the RAP ID is Os06t0133000-01 which should be entered as OS06G013300 in the search box.

3. For searches in Shuhui498 (*indica*) the gene ID should start OsR498 (e.g. OsR498G0100000700.01): **e.g an example gene ID in 93-11 (*indica*) is BGIOSGA014191.**

5. Note that gene annotation may differ between databases. If you can't find a gene using the gene ID try searching for the search for KASP by genome position instead.

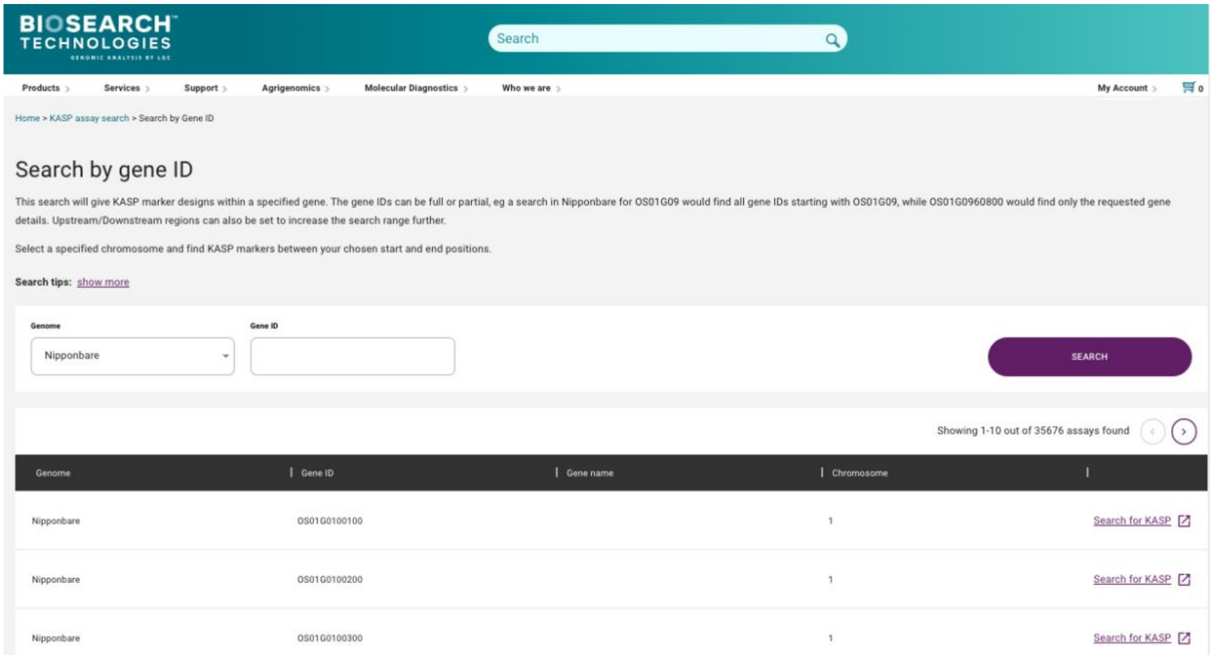

<u>Search Gene ID and flanking regions</u>

Search for KASP markers within a specified gene and up to 10 kb either side of that gene. The gene IDs entered can be full or partial, e.g. a search in Nipponbare for OS01G09 would find all gene IDs starting with OS01G09, while OS01G0960800 would find only the requested gene details. Upstream/Downstream regions can also be set to increase the search range further.

Search tips:
Please be cautious that the search tool is case-sensitive and that the gene IDs used for the search may require different formats depending on the Genome. Also, make sure that you are not including spaces in the text typed. Please carefully read the instructions below.

1. If the gene ID or name gives no hits, refer to the Rice Annotation Project database (rapdb.dna.affrc.go.jp) and use the keyword search to find the RAP gene ID corresponding to the gene name that you want to look up.

2. For searches in Nipponbare (*japonica*) the gene ID should start with OS. Enter the gene ID in the box using only uppercase letters and always use a G as the fifth character and ignore any dash and further digits: **e.g for the gene known as Waxy the RAP ID is Os06t0133000-01 which should be entered as OS06G013300 in the search box.**

3. For searches in Shuhui498 (R498, *indica*) the gene ID should start OsR498 (e.g. OsR498G0100000700.01) and the s must be lower case: **e.g an example gene ID in 93-11 (*indica*) is BGIOSGA014191**

5. Note that gene annotation may differ between databases. If you can't find a gene using the gene ID try search by genome position instead.

6. When the SNP or InDel is not located within a known gene, the gene ID is shown as "undefined".

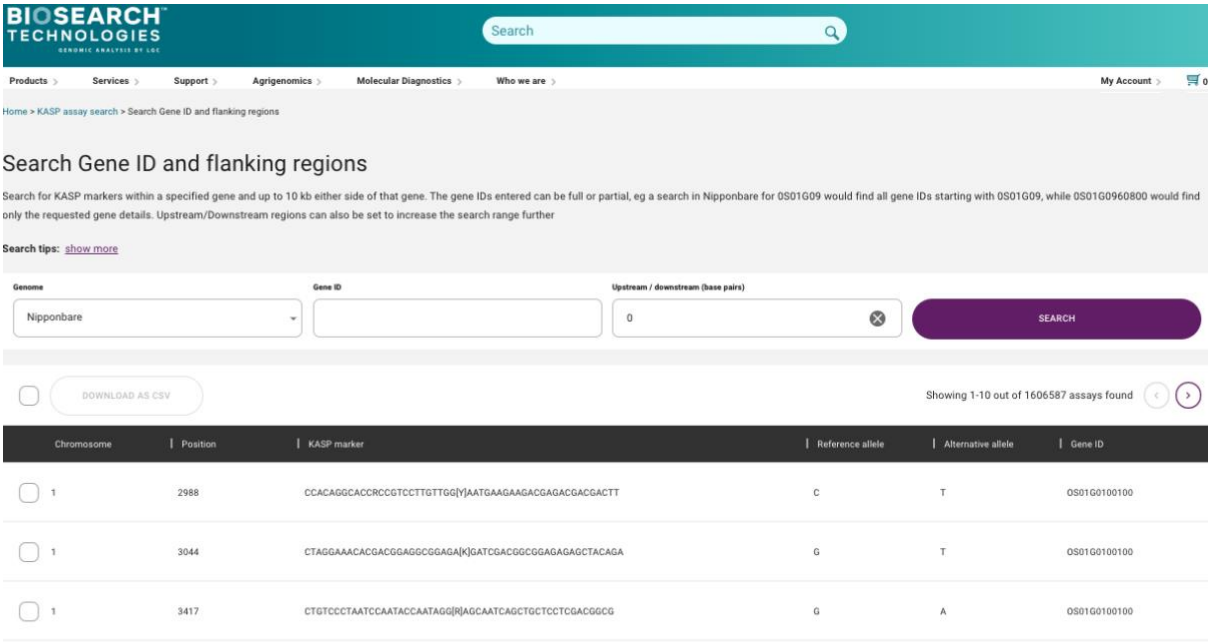

Search by microsatellite ID or C6AIR SNP ID

Search using the names of published SSR (simple sequence repeat) microsatellite markers or SNPs listed in the Cornell 6K SNP array (C6AIR) to find assays which are either located between them or within a specified distance from them. If you only enter one marker it is suggested that you start with a minimum of 2000 bp upstream and downstream. You can also search the region between two named markers (e.g. RM24 and RM129) if they are on the same chromosome. Upstream/Downstream regions can also be set to increase the search range further.

Search tips:
Please be cautious that the search tool is case-sensitive and that the gene IDs used for the search may require different formats depending on the Genome. Also, make sure that you are not including spaces in the text typed. Please carefully read the instructions below.

1. If the search does not work with two named markers, start with searching for each marker separately first, **e.g. AUT10008 and include 2000 bp up and downstream.**

2. **Cornell 6K SNP (C6AIR) IDs should be given as published by Thompson et al. (2017. Rice 10:40, doi: 10.1186/s12284-017-0181-2) and listed in the Additional file 6 TableS2.**

3. Note that this tool includes only a selection of published SSRs and does not include any published assay ID names of KASP. If marker IDs have no hits over 10,000 bp then try the genome position search instead.

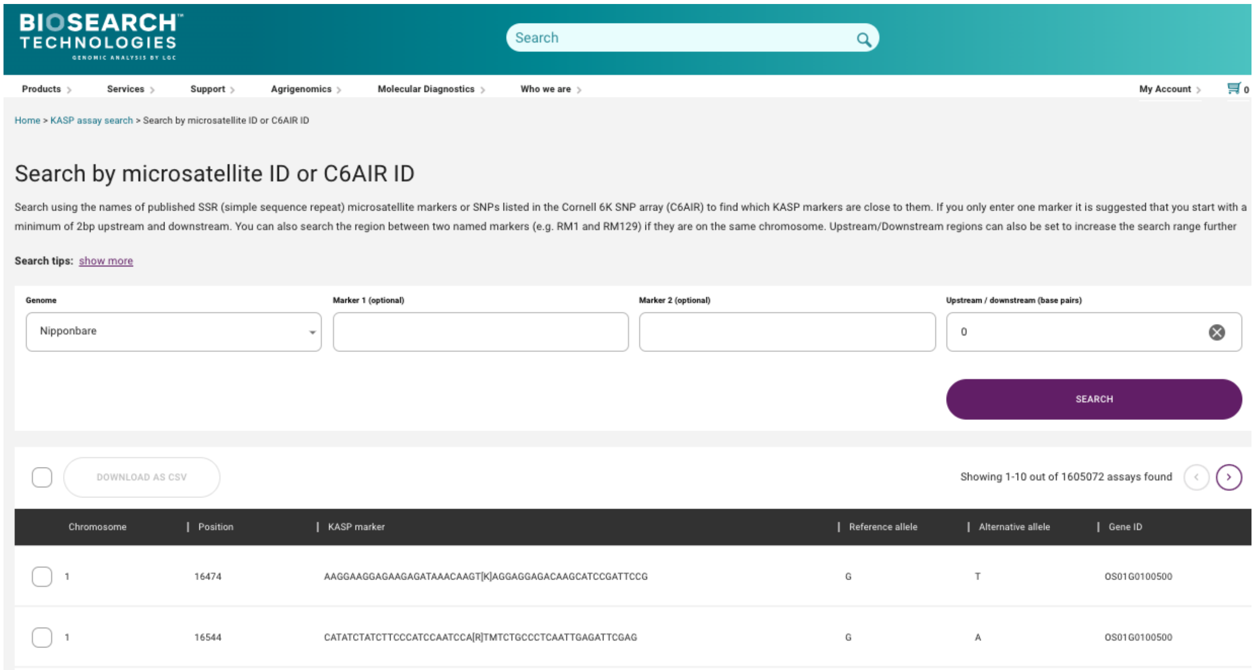

Search by microsatellite ID or C6AIR SNP ID from position on chromosome

Search for KASP markers on a specified chromosome located at a specified SSR (simple sequence repeat) or C6AIR (Cornell 6K SNP array) and up to 10 kb either side of that marker.

Search tips:
Warning: This search only shows KASP that target the same SNP as a C6AIR marker or target a SNP within 2000 bp of a microsatellite. Any other KASP designs in the region, such as those targeting a gene are not shown in this search.

If you have a marker name for a C6AIR SNP:

1. Select C6AIR for "Marker type".

2. Type in the "Marker ID" field (for example id12010130).
   - **C6AIR ID should be given as published by Thompson et al. (2017. Rice 10:40, doi: 10.1186/s12284-017-0181-2) and listed in the Additional file 6 TableS2.**
   - **Any dash (-) symbol must be replaced by N (for example SNP-12_25758809 should be searched typing SNPN12_25758809).**
3. Select "Genome" as Any.
4. Press "Search" to find the location of this marker in any of the three genomes.
5. From the list of results, click on the relevant genome to open a search starting from that marker position (Search by genome position), which allows broadening the search across a wider region.

If you have a marker name for a Cornell microsatellite:

1. Select SSR for "Marker type".
2. Type in RM1 for "Marker ID".
3. Select "Genome" as Any.
4. Press "Search" to find the location of this marker in any of the three genomes.
5. From the list of results, click on the relevant genome to open a search starting from that microsatellite position (Search by genome position). Note that there are no KASP within a simple sequence repeat, so we recommend searching the flanking region by decreasing the start position by -1000 and increasing the end position by +1000 in the Search by genome position.

Use a search similar to the above example if you know the region of a chromosome and want to find KASP markers close to previously published SSR markers:

1. Select SSR for "Marker type".
2. Leave "Marker ID" blank.
3. Select Nipponbare for "Genome".
4. Enter 1 for "Chromosome".
5. Select 1 for "Start position" and 1000000 for "End position".
6. Scroll down the list of markers and click on any one to download or to broaden the search across wider region (Search by genome position).

Supplementary File S3 Users' guide to the 'Rice Assay Search Tool' for searching the database containing the BU_LGC_plus Rice genotyping panel of KASP designs

Steele K.A., Quinton-Tulloch M., Vyas D. & Witcombe J.R., 2024, G3: Genes|Genomes|Genetics

Search by microsatellite ID or C6AIR ID from list

Search for KASP markers on a specified chromosome located at a specified SSR or C6AIR and up to 10 kb either side of that marker.

Type	Gene ID	Chromosome	Upstream / downstream (base pairs)
SSR			

SEARCH

Showing 1-20 out of 46 assays found

Marker ID	93-11 Chr.	Start	End	Nipponbare Chr.	Start	End	Shuhui_498 Chr.	Start	End
AUT10010	17	39235	45904	24	9631	16415	9	20796	18599
AUT10169	6	18599	18599	27	45904	50963	-1	-1	-1
AUT10124	9	16627	16627	-1	-1	-1	11	23340	95554
AUT10012	10	61391	16415	22	45904	50364	16	50364	20796
AUT10020	-1	-1	-1	19	93457	61391	7	92771	61391
AUT10045	-1	-1	-1	27	9631	74875	1	83676	16415
AUT10168	-1	-1	-1	25	93457	83676	10	83676	39235
AUT10057	3	16415	10708	20	50963	3398	3	39235	50963
AUT10205	22	45904	20796	14	23340	74875	13	18599	39235
AUT10105	27	93046	50364	8	16415	28200	18	20079	16415
AUT10193	1	93457	23340	10	23340	45904	23	92771	43359
AUT10026	23	3398	13671	17	9631	23340	5	92771	18599
AUT10121	26	20079	4339	15	20796	93046	2	9631	20796
AUT10179	20	92771	50963	26	18599	92771	7	61391	23340
AUT10121	13	9631	20079	16	23340	93457	6	28200	61391
AUT10060	18	50963	74875	14	13671	93457	22	92771	61391

Search by genome position

Enter a specified chromosome (do not include 0 for chromosomes 1-9) and find KASP markers between your chosen start and end positions.

Search tips:
1. Try searching with the same approximate positions in more than one genome to locate all available KASP in each genome - but note that the positions do not match up exactly between the different reference genomes.

7. 2. Subtract ≥1000 bp from the first co-ordinate and add ≥1000 to the second co-ordinate to widen the search.
8. 3. Note that leaving the chromosome box blank will give results for all chromosomes.
9. 4. If you get no hits with the position but you know the nearest gene ID try the Gene ID search instead.

Supplementary File S3 Users' guide to the 'Rice Assay Search Tool' for searching the database containing the BU_LGC_plus Rice genotyping panel of KASP designs

Steele K.A., Quinton-Tulloch M., Vyas D. & Witcombe J.R., 2024, G3: Genes|Genomes|Genetics

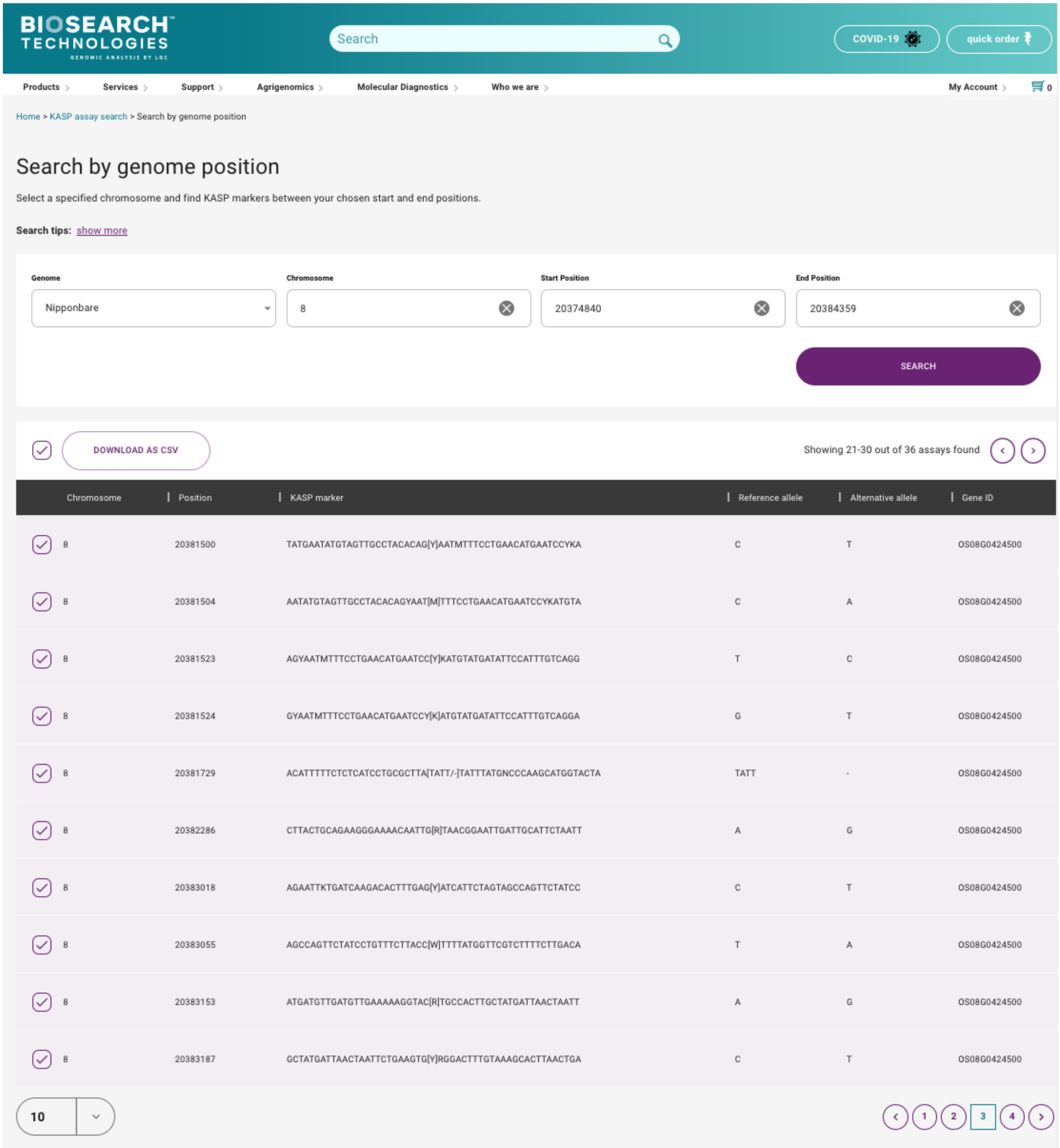

Chromosome	Position	KASP marker	Reference allele	Alternative allele	Gene ID
8	20381500	TATGAATATGTAGTTGCCTACACAG[Y]AATMTTTCCTGAACATGAATCCYKA	C	T	OS08G0424500
8	20381504	AATATGTAGTTGCCTACACAGYAAT[M]TTTCCTGAACATGAATCCYKATGTA	C	A	OS08G0424500
8	20381523	AGYAATMTTTCCTGAACATGAATCC[Y]KATGTATGATATTCCATTTGTCAGG	T	C	OS08G0424500
8	20381524	GYAATMTTTCCTGAACATGAATCCY[K]ATGTATGATATTCCATTTGTCAGGA	G	T	OS08G0424500
8	20381729	ACATTTTTCTCTCATCCTGCGCTTA[TATT/-]TATTTATGNCCCAAGCATGGTACTA	TATT	-	OS08G0424500
8	20382286	CTTACTGCAGAAGGGAAAACAATTG[R]TAACGGAATTGATTGCATTCTAATT	A	G	OS08G0424500
8	20383018	AGAATTKTGATCAAGACACTTTGAG[Y]ATCATTCTAGTAGCCAGTTCTATCC	C	T	OS08G0424500
8	20383055	AGCCAGTTCTATCCTGTTTCTTACC[W]TTTTATGGTTCGTCTTTTCTTGACA	T	A	OS08G0424500
8	20383153	ATGATGTTGATGTTGAAAAAGGTAC[R]TGCCACTTGCTATGATTAACTAATT	A	G	OS08G0424500
8	20383187	GCTATGATTAACTAATTCTGAAGTG[Y]RGGACTTTGTAAAGCACTTAACTGA	C	T	OS08G0424500
