# Supplementary material for: Thousands of trait-specific KASP markers designed for diverse breeding applications in rice (Oryza sativa)
Source: G3 (Bethesda). 2024 Nov 1;15(1):jkae251. doi: 10.1093/g3journal/jkae251 (PMC11708223; doi:10.1093/g3journal/jkae251)
Supplement: jkae251_Supplementary_Data [file jkae251_supplementary_data.zip › Supplemental_Material_Legends_G3-2024-405461.docx]

**Supplementary Resources**

**File S1.**

**Note A.** Methods for sequencing two previously unpublished genomes

**Figure A**. Success rates from genotyping of 178 samples with 4,000 KASP markers (a) Percentage of samples with genotype calls for each marker; (b) Percentage of markers with genotype calls for each sample.

**Note B.** Naming of KASP markers

**Figure B.** Relationship between number of degenerate nucleotides in KASP design and the number of possible designs (predicted), distance between KASP designs (predicted)and percentage of successful assays (actual). Data used for this plot are presented in Table 3.

**File S2.** Excel file with 13 Supplementary tables:

**Table A**. Details of 129 rice genomes and their EBI Accession Numbers

**Table B**. Summary of Bangor University (BU) and 3k RGP genomes data

**Table C** Location of 5274 C6IAR in three reference genomes

**Table D** Filtering outcomes of C6IAR for KASP designs

**Table E** Locations of 17,924 Gramene microsatellite markers in three reference genomes

**Table F**. Details of 5028 KASP designs with associated target genes or markers selected for validation tests in 178 rice genotypes

**Table G**. The number (1-5) of KASP designs included for 1079 target genes or markers

**Table H**. Details of 178 varieties used for genotyping and population analysis

**Table I**. Summary of validation data for 3366 KASP

**Table J**. Genotype calls of 178 varieties tested with 3366 KASP (599,148 data points)

**Table K** 4985 KASP design targets with their locations in R498 and Nipponbare and nearest SSR markers

**Table L**. Comparison between sequenced and genotyped alleles (sub-set of 43,758 data points) for 13 lines and 3366 markers

**Table M**. Example output files for two searches for KASP assays using the BU/LGC+ Rice KASP Database

**File S3.** Users’ guide to the ‘Rice Assay Search Tool’ for searching the database containing the BU_LGC_plus Rice genotyping panel of KASP designs
